# A panel of recombinant *Leishmania donovani* cell surface and secreted proteins identifies LdBPK_323600.1 as a serological marker of symptomatic infection

**DOI:** 10.1128/mbio.00859-24

**Published:** 2024-04-19

**Authors:** Adam J. Roberts, Han Boon Ong, Simon Clare, Cordelia Brandt, Katherine Harcourt, Yegnasew Takele, Prakash Ghosh, Angela Toepp, Max Waugh, Daniel Matano, Anna Färnert, Emily Adams, Javier Moreno, Margaret Mbuchi, Christine Petersen, Dinesh Mondal, Pascale Kropf, Gavin J. Wright

**Affiliations:** 1 Hull York Medical School, University of York, Heslington, York, United Kingdom; 2 Cell Surface Signalling Laboratory, Wellcome Sanger Institute, Cambridge, United Kingdom; 3 Pathogen Laboratory Support, Wellcome Sanger Institute, Cambridge, United Kingdom; 4 Department of Infectious Disease, Imperial College London, London, United Kingdom; 5 International Centre for Diarrhoeal Disease Research, Dhaka, Bangladesh; 6 College of Public Health, University of Iowa, Iowa City, USA; 7 Centre for Clinical Research, Kenya Medical Research Institute (KEMRI), Nairobi, Kenya; 8 Department of Medicine Solna and Center for Molecular Medicine, Division of Infectious Diseases, Karolinska Institutet, Stockholm, Sweden; 9 Department of Infectious Diseases, Karolinska University Hospital, Stockholm, Sweden; 10 Centre for Drugs and Diagnostics, Liverpool School of Tropical Medicine, Liverpool, United Kingdom; 11 WHO Collaborating Centre for Leishmaniasis, National Centre for Microbiology, Instituto de Salud Carlos III, CIBER de Enfermedades Infecciosas—CIBERINFEC, Madrid, Spain; 12 Department of Biology and York, Biomedical Research Institute, University of York, York, United Kingdom; NIAID/NIH, Rockville, Maryland, USA

**Keywords:** serology, proteins, visceral leishmaniasis, ELISA, *Leishmania donovani*, recombinant proteins

## Abstract

Visceral leishmaniasis is a deadly infectious disease and is one of the world’s major neglected health problems. Because the symptoms of infection are similar to other endemic diseases, accurate diagnosis is crucial for appropriate treatment. Definitive diagnosis using splenic or bone marrow aspirates is highly invasive, and so, serological assays are preferred, including the direct agglutination test (DAT) or rK39 strip test. These tests, however, are either difficult to perform in the field (DAT) or lack specificity in some endemic regions (rK39), making the development of new tests a research priority. The availability of *Leishmania* spp. genomes presents an opportunity to identify new diagnostic targets. Here, we use genome data and a mammalian protein expression system to create a panel of 93 proteins consisting of the extracellular ectodomains of the *Leishmania donovani* cell surface and secreted proteins. We use these panel and sera from murine experimental infection models and natural human and canine infections to identify new candidates for serological diagnosis. We observed a concordance between the most immunoreactive antigens in different host species and transmission settings. The antigen encoded by the *LdBPK_323600.1* gene can diagnose *Leishmania* infections with high sensitivity and specificity in patient cohorts from different endemic regions including Bangladesh and Ethiopia. In longitudinal sampling of treated patients, we observed reductions in immunoreactivity to LdBPK_323600.1 suggesting it could be used to diagnose treatment success. In summary, we have identified new antigens that could contribute to improved serological diagnostic tests to help control the impact of this deadly tropical infectious disease.

**IMPORTANCE:**

Visceral leishmaniasis is fatal if left untreated with patients often displaying mild and non-specific symptoms during the early stages of infection making accurate diagnosis important. Current methods for diagnosis require highly trained medical staff to perform highly invasive biopsies of the liver or bone marrow which pose risks to the patient. Less invasive molecular tests are available but can suffer from regional variations in their ability to accurately diagnose an infection. To identify new diagnostic markers of visceral leishmaniasis, we produced and tested a panel of 93 proteins identified from the genome of the parasite responsible for this disease. We found that the pattern of host antibody reactivity to these proteins was broadly consistent across naturally acquired infections in both human patients and dogs, as well as experimental rodent infections. We identified a new protein called LdBPK_323600.1 that could accurately diagnose visceral leishmaniasis infections in humans.

## INTRODUCTION

Visceral leishmaniasis (VL) is a vector-borne tropical disease caused by the protozoan parasites *Leishmania donovani* and *Leishmania infantum* that is usually fatal if left untreated. The disease is primarily transmitted through the bite of an infected phlebotomine sand fly, and there are an estimated 50,000 to 90,000 new annual cases of VL worldwide, a number that is greater than the official figures reported by the World Health Organization (WHO) (1), due to underreporting. VL disproportionately affects people living in poverty in tropical and developing countries, with more than 95% of the reported cases in 2018 occurring in 10 countries: Brazil, China, Ethiopia, India, Iraq, Kenya, Nepal, Somalia, South Sudan, and Sudan (2). Clinically, enlargement of the spleen and liver together with anemia is a typical characteristic of late-stage VL, while early symptoms include irregular bouts of fever and weight loss that can be easily misdiagnosed as other endemic diseases such as malaria or enteric fever (3). Diagnostic tests provide specific, rapid, and accurate detection, especially during the early stages of infection and are crucial for effective treatment, surveillance, and control of VL.

Historically, VL is diagnosed by parasitological analyses of smear preparations from spleen, lymph nodes, or bone marrow aspirates. While this is often regarded as the gold standard for VL diagnosis and offers a high degree of specificity, it is laborious and suffers from variations in sensitivity. Organ aspirations are invasive and require highly trained medical professionals to avoid complications and possible death (3). Molecular diagnostic methods such as PCR and real-time PCR offer high sensitivities and specificities but require highly trained operators and can be prohibitively costly for many endemic countries.

The need for alternative diagnostic methods that are safer and more deployable led to the development of two serological field tests: the direct agglutination test (DAT) and recombinant K39 antigen immunochromatographic strip test (rK39 ICT). Because both tests detect host antibody responses which persist, they do not distinguish between patients with current infections and those who have recovered; consequently, these methods cannot be used to diagnose relapses or re-infections. DAT uses freeze-dried parasites as antigens to measure antibody titers present in VL patients (4). The method is cumbersome to perform, usually requiring an overnight incubation step, and results can vary depending on the experience of trained operators (5). More recently, the rK39 ICT gained popularity due to its high specificity for VL and its ease of use in remote areas without requirement for additional equipment while still providing rapid results (6). rK39 ICT is a lateral flow “dipstick” assay using a recombinant form of the *L. infantum* kinesin-like K39 antigen, and while the performance with respect to sensitivity of rK39 ICT is favorable in many clinical settings, high false negative results of rK39 ICT have been reported (7, 8). Importantly, the sensitivity of the rK39 assay can also be reduced in regions endemic for other infectious diseases including HIV and other parasitic infections (9). Several studies have advocated its use in combination with other methods such as DAT to improve its diagnostic power (9–11).

*L. donovani*, and in particular *L. infantum*, have broad host ranges, infecting a range of domestic and wild animals that can act as reservoirs for zoonotic transmission to humans. Companion animals, and especially dogs, are particularly important because of their proximity to humans. These infections can often be asymptomatic (12–14), and while the rK39 assay has been used for the diagnosis of canine leishmaniasis (CanL), limitations have been reported (15, 16). The absence of a highly effective vaccine means that surveillance and epidemiological studies are critical for the control and prevention of outbreaks of the disease which require reliable diagnostic assays (9). Further improvements to serological diagnosis will therefore benefit from an increased repertoire of specific antigens that can be used in combination for VL diagnosis.

Extracellular parasite antigens can be informative early immunodiagnostic markers as they are directly exposed to the host humoral immune system, and the sequencing of the *L. donovani* (17) and *L. infantum* (18 ) genomes revealed a large number of new candidate antigens. Despite their value, however, extracellular proteins can be challenging to express in a recombinant form because they contain structurally important post-translational modifications such as disulfide bonds that are required for correct folding to create informative conformational antibody epitopes. To address this, we have developed an approach based on a mammalian expression system to systematically express large panels of recombinant proteins that comprise the extracellular domains of cell surface and secreted parasite proteins that retain their binding activity and immunogenicity for several different parasites (19–24).

Here, we have compiled a library of *L. donovani* proteins predicted to be secreted or displayed at the parasite cell surface and determined their immunoreactivity to sera raised against *L. donovani* and *L. infantum*. Our protein library was screened against sera from experimental infections performed in mice, natural infections in dogs, and human infections from several different endemic regions. Our findings have yielded a list of potential antigens that could be progressed as diagnostic serological markers for VL infections.

## RESULTS

### A library of recombinant *L. donovani* cell surface and secreted proteins

**Fig 1:**
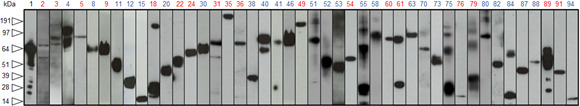
A library of recombinant *L. donovani* cell surface and secreted proteins. Recombinant *L. donovani* proteins were purified from spent cell culture supernatant; expression levels were normalized, resolved by SDS-PAGE under reducing conditions, and blotted; and biotinylated proteins were detected using streptavidin-horseradish peroxidase. Proteins are systematically numbered for convenience (Table S1); blue labels indicate proteins expressed with biotin and his-tag only and red labels with the additional rat Cd4 tag.

To identify cell surface and secreted proteins that could be valuable serological diagnostic markers for VL, we compiled a list of proteins based on the *L. donovani* (strain BPK282A1) genome using data from both published mass spectrometry analyses (25–27) and *in silico* prediction tools on TriTryDB (28). Amino acid sequences of these proteins were analyzed for the presence of transmembrane-spanning regions, glycosylphosphatidylinositol anchors, and signal peptides using computational prediction tools (29–31). A collection of 93 plasmids encoding the ectodomains of *L. donovani* cell surface and secreted proteins was compiled which were labeled with a “D” and consecutively numbered for convenience (Table S1). Each plasmid was used to transfect HEK293 cells to increase the chances that structurally critical post-translational modifications such as disulfide bonds were faithfully added. Initially, all proteins were expressed containing a C-terminal peptide encoding a His-tag and a biotin acceptor sequence that can be enzymatically biotinylated when co-transfected with the BirA biotin ligase (32). For those proteins that were not expressed highly, we included an additional C-terminal rat Cd4 domains 3 and 4 tag which can improve protein expression (33). The expression levels of each protein were quantified by enzyme-linked immunosorbent assay (ELISA), and their integrity was determined by western blotting (Fig. 1). In total, 45 (48%) of the proteins could be detectably expressed including extremely large proteins such as LdBPK_292300.1 (D35, ~213 kDa) (Fig. 1). Most proteins resolved as a broad band around the predicted molecular mass suggesting that each preparation contained a range of glycoforms as expected. In some cases, several bands were detected suggesting these proteins were proteolytically processed which is occasionally observed using this expression system (34).

### Recombinant *L. donovani* proteins were immunoreactive to sera from infected hosts

**Fig 2:**
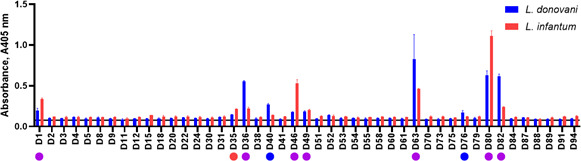
A library of recombinant cell surface and secreted proteins systematically identifies immunoreactive *Leishmania donovani* and *Leishmania infantum* proteins using sera from experimentally infected mice. The library of *L. donovani* proteins were expressed as biotinylated proteins and immobilized on streptavidin-coated plates. Leishmania protein arrays were probed with sera pooled from five mice experimentally infected with *L. donovani* or *L. infantum* and immunoreactivity to each antigen quantified by ELISA using an anti-mouse IgG antibody. Data points represent mean ± SD of triplicate measurements for infected sera; the solid line represents the mean of the naive serum measurements against the whole library. Immunoreactivity was considered positive if the mean was greater than 2 SD above the mean of all controls (solid line). Proteins are marked by circles indicating if it was reactive with *L. donovani* (blue), *L. infantum* (red), or both (purple).

To confirm if the purified recombinant ectodomains could be used for serological diagnosis of visceral leishmaniasis, we tested the protein library by ELISA against convalescent serum from a murine *L. donovani* infection model (35). Sera that had been collected from mice which had been infected for 20 weeks demonstrated clear immunoreactivity to nine proteins: D1, 36, 40, 46, 49, 63, 76, 80, and 82 compared with sera from uninfected mice (Fig. 2). Importantly, the immunoreactivity was shown to be heat labile demonstrating that most antibody recognition was dependent upon the presence of conformational epitopes suggesting that these protein ectodomains are likely to be correctly folded (Fig. S1). To confirm and extend our findings, we also tested sera from mice that had been chronically infected with the related parasite, *L. infantum*. We observed that the immunoreactivity to *L. infantum* sera across our antigen panel was broadly similar, although two (D40 and D82) of the antigens exhibited much reduced immunoreactivity while D1, D35, D46, and especially D80 demonstrated greater immunoreactivity (Fig. 2). Immunoreactive antigens from both infections differed in the magnitude of immunoreactivity, with the highest responses observed against D80 (a “hypothetical” protein) and D63, the homolog of the *Trypanosoma cruzi* GP72 (36) and *T. brucei* FLA1 proteins (37).

### Kinetic profiling of antigen reactivity in experimental infection models

**Fig 3:**
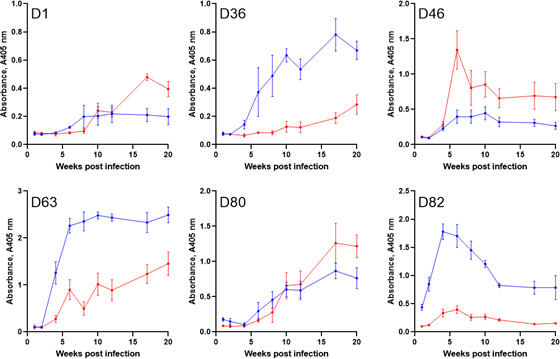
Kinetics of the serological response to immunoreactive antigens in experimentally infected mice. The indicated *L. donovani* antigens were expressed as biotinylated proteins and captured within individual wells of a streptavidin-coated microtiter plate. The time-resolved immunoreactivity for each protein was quantified using sera taken from groups of five BALB/c mice experimentally infected with either *L. donovani* (blue) or *L. infantum* (red) at regular intervals over a 20-week infection protocol. Data points represent means ± SD; *n* = 5. Note that one mouse infected with *L. donovani* was removed from the experiment from week 16 onwards.

To determine if immunoreactive antigens could be indicators of disease progression, longitudinal serum samples from both *L. donovani* and *L. infantum* infections of BALB/c mice (35) were systematically tested against the protein library. We observed that the serological responses to *L. donovani* were remarkably consistent between individual mice and agreed with the results from the pooled sera (Fig. 3). For example, D63 (FLA1) was again the most immunoreactive antigen, and the antibody responses to the other proteins retained their same relative magnitude. High-titer antibody responses to both D63 and D82 were observed as early as 4 weeks post-infection, especially for D82, where robust responses were detected even at the earliest time point at 1 week post-infection. Reactivity to three other antigens (D1, D36, and D80) was only apparent 4 to 6 weeks post-infection, suggesting a delayed antibody response to these markers. As expected, the antibody titers were cumulative during the infection with the remarkable exception of D82, where the titers, after peaking at 4 weeks post-infection, reduced until plateauing at 12 weeks. The responses to the proteins in sera from mice infected with *L. infantum* broadly matched those responses to *L. donovani* with the exception that immunoreactivity to D36 and D63 took noticeably longer to acquire and was of a lower titer. Similarly, although of lower amplitude, the kinetics of immunoreactivity to D82 in *L. infantum* infections were similar to those in *L. donovani*, showing a peak of reactivity at 5 weeks post-infection before declining (Fig. 3). In summary, our results provided indications that the pattern of antibody responses to defined antigens could be used to estimate if an infection was at an early or late stage.

### Identification of serological markers for canine leishmaniasis

**Fig 4:**
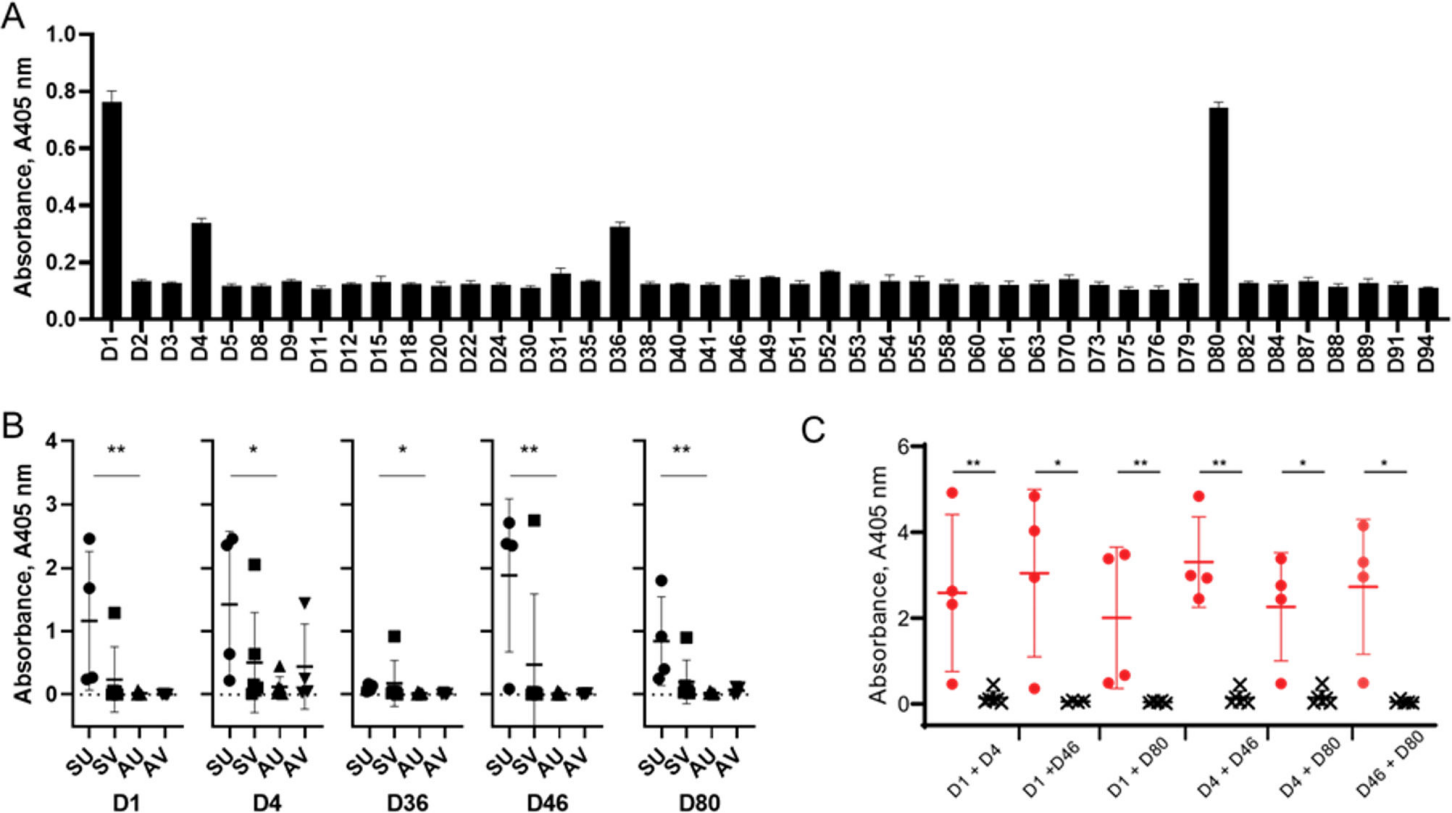
Serological markers of canine leishmaniasis can be used to distinguish progressive disease. (**A**) Pooled serum from Spanish dogs naturally infected with *L. infantum* showed a similar pattern of immunoreactivity to serum from experimentally infected mice. The library of soluble recombinant *L. donovani* proteins was expressed as biotinylated proteins and immobilized in individual wells of streptavidin-coated microtiter plates. The protein arrays were probed for binding with pooled serum collected from *L. infantum*-infected dogs and immunoreactivity quantified by ELISA. Data points represent means ± SD, *n* = 3. (**B**) Experimental immunotherapy of the mothers significantly reduces the titer of antibodies in canine visceral leishmaniasis. Individual serum samples from dogs that had contracted leishmaniasis through maternal transmission were screened against a subset of the library by ELISA. Serum samples were classified from dogs that showed symptoms of disease (S) or were asymptomatic (A) and if they had received the therapeutic vaccine Leish-Tec (V) or were unvaccinated (U). Almost all seropositive dogs were from the symptomatic group (compare SU and SV to AU and AV), and where vaccinated groups still developed symptoms, they generally exhibited reduced antibody titers (compare SU and SV). Data points represent individual animals; bar indicates mean ± SD. The Mann-Whitney test was used define significant differences between the symptomatic and asymptomatic animals that had either received a therapeutic vaccination or not. (**C**) Combining the immunoreactivity signals from multiple antigens improves the ability to distinguish between symptomatic (red circles) and asymptomatic (black crosses) canine infections. Data points represent summed ELISA readings from the indicated protein pairs for each individual. Statistical significance was inferred using the Mann-Whitney test. Significance is indicated in B and C as *P > 0.05 and **P > 0.01.

*L. infantum* transmission in dogs poses a significant threat to human health because they are known reservoirs for zoonotic transmission and are popular companion animals (38). To determine if the proposed recombinant target proteins can also be used as serological markers for canine leishmaniasis, pooled sera from Spanish dogs that were naturally infected with *L. infantum* were tested against our protein library. Consistent with our results from mouse sera, proteins D1, D36, and D80 were highly immunoreactive (Fig. 4A); despite this similar reactivity toward a subset of antigens in the host species, differences in which antigens cross-reacted with the sera were observed for D4, which became immunoreactive with the pooled canine sera, and immunoreactivity against D25, 46, 63, and 82 was not detectable.

There is increasing evidence of transplacental transmission of *Leishmania* from mother to pups in dogs (39–41). To extend our findings, we screened sera from American pups that had maternally contracted an *L. infantum* infection against the protein library to determine if it could be used to diagnose natural vertical transmission. Samples were further categorized into those animals who had either been administered with a therapeutic vaccine or left unvaccinated and then those that developed symptoms of infection from those that were asymptomatic (Table S2) (40). Using a subpanel of proteins from the library, we observed that the same four antigens (D1, D4, D36, and D80) were again immunoreactive (Fig. 4B ; Table S3). Due to its immunoreactivity with the experimental murine infections, we also included D46 to be positive in these animals but not the pooled samples, a difference which may be due to the transplacental route of infection (Fig. 4B). Importantly, clear immunoreactivity was observed in several dogs that had developed symptomatic disease compared with those that were asymptomatic (Fig. 4B). Interestingly, we found that immunoreactivity was reduced in dogs that had been vaccinated but nevertheless exhibited some symptoms of infection (Fig. 4B). We noticed that no single antigen was universally positive in all animals exhibiting symptoms suggesting that combining their immunoreactivity could lead to improved diagnosis. Indeed, when we combined the immunoreactivity from pairs of antigens, this led to improved diagnostic sensitivity with the best result obtained when combining D4 and D46 (Fig. 4C).

### A general concordance of immunoreactive antigens to Leishmania infection in humans with mice and dogs

**Fig 5:**
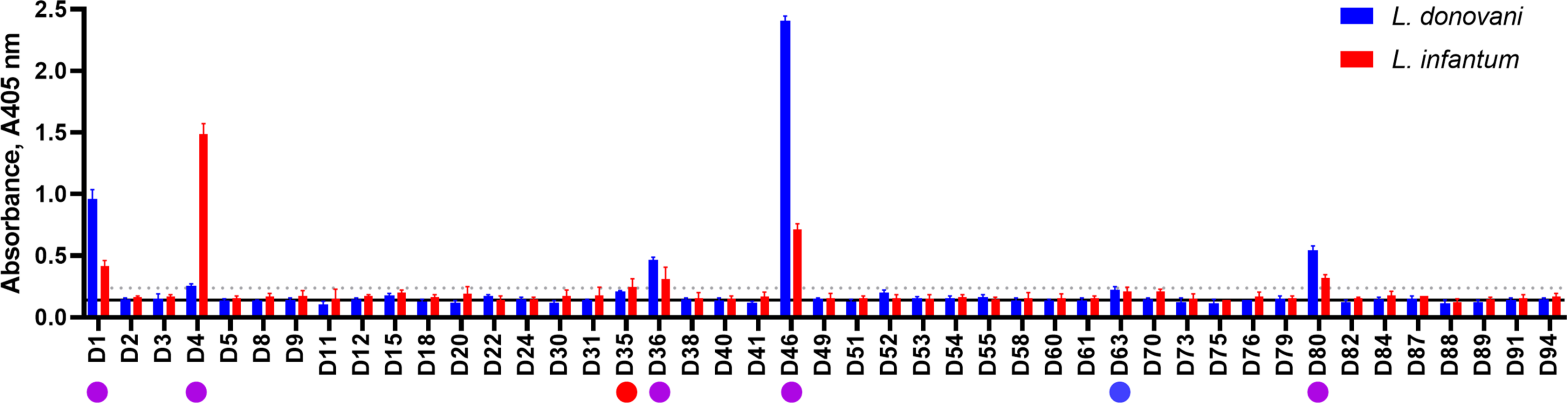
Systematic screening of sera from *L. donovani-* or *L. infantum*-infected patients revealed a concordance of immunoreactive antigens in human, canine, and murine hosts. Pooled sera from diagnosed human *L. donovani* (Ethiopia) and *L. infantum* (Spain) infections were probed against the protein library and immunoreactivity quantified. Bars represent means ± SD; *n* = 3. The solid line represents the mean of pooled naive serum against the whole protein library; the dashed line indicates this mean measurement + 5 SD. A total of 7 proteins had higher immunoreactivity greater than 5 SD (dotted line) above the naive serum mean (solid line) in *L. donovani* or *L. infantum* patient sera. Proteins are marked by circles indicating if it was reactive with *L. donovani* (blue), *L. infantum* (red), or both (purple).

To determine if our panel of proteins could be used to diagnose sand fly-transmitted *Leishmania* infections in humans, we first tested pooled sera from humans with diagnosed visceral Leishmania infections to both *L. donovani* and *L. infantum* from Spain (9). We observed that five (D1, D4, D36, D46, and D80) of the nine proteins that had been previously identified as highly immunoreactive were consistently immunoreactive in human populations infected with either *L. donovani* or *L. infantum* with means greater than 1.5 SD above the mean of the controls (Fig. 5). As with murine experimental infections, we observed differences in antigen reactivity with sera from *L. donovani-* and *L. infantum*-infected humans with the most immunoreactive antigens being D46 in *L. donovani* and D4 in *L. infantum* infections.

### Immunoreactivity with the LdBPK_323600.1 ectodomain is diagnostic of symptomatic infection in VL patients

**Fig 6:**
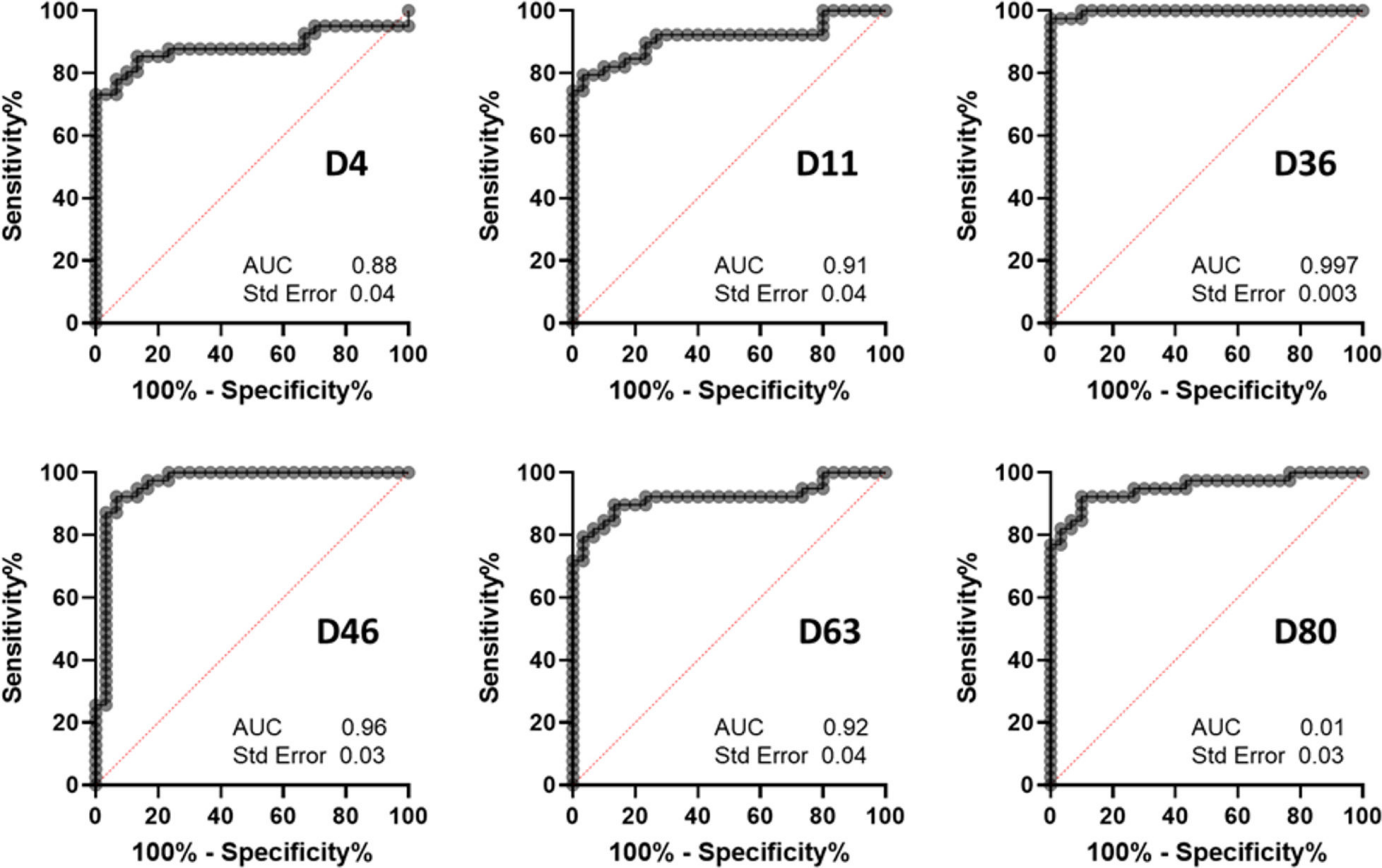
Seroimmunoreactivity to recombinant D36 (LdBPK_323600.1)-classified visceral leishmaniasis patients from Bangladesh with high sensitivity and specificity. The named recombinant biotinylated *L. donovani* proteins were immobilized in streptavidin-coated microtiter plates, and immunoreactivity to patient serum from either diagnosed symptomatic (*n* = 39) or diagnosed uninfected endemic (*n* = 30) controls was quantified by ELISA. Data are presented as receiver-operator curves where the area under the curve (AUC) and standard errors are shown for each antigen.

To determine if the new antigens could be used for serological screening of visceral infections in humans, we first used a serum collection containing rK39-diagnosed positive visceral leishmaniasis patients (Table 1; Table S4) collected in Bangladesh with regionally matched diagnosed negative control sera (5). We focused on five proteins: D4, 36, 46, 63, and 80, all of which had previously been shown to be immunoreactive and could be expressed at high levels, as well as a sixth protein, D11, which, when administered as a vaccine, led to reduced disease severity in a murine model (42). We observed that the immunoreactivity to several proteins could classify diagnosed infections from controls with high specificity and sensitivity with D36 (LdBPK_323600.1) performing the best of all (97.44% sensitivity and 96.67% specificity [Fig. 6]) and with comparable accuracy to the rK39 test and DAT tests.

**TABLE 1:** Summary of diagnostic test results of symptomatic VL patients by region

Country of origin	*n*	Samples with missing metadata	rK39 positive	rK28 positive	DAT positive
Kenya	45	1	90.9%	88.6%	95.5%
Bangladesh	40	0	100.0%	N/A	100.0%

**Fig 7:**
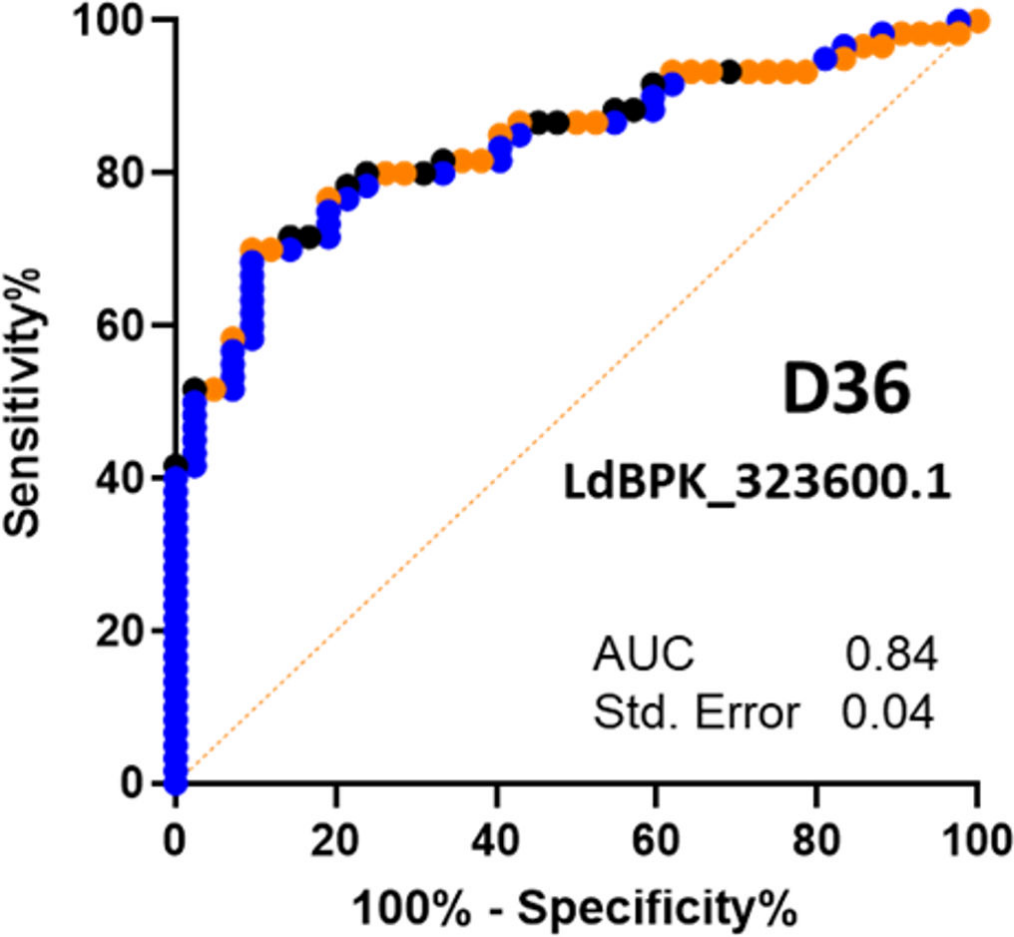
Immunoreactivity to LdBPK_323600.1 (D36)-classified visceral leishmaniasis in Kenyan patients with high sensitivity and specificity. Receiver operator curve showing the immunoreactivity measured by ELISA to LdBPK_32600.1 (D36) in diagnosed visceral leishmaniasis patients (*n* = 60, blue). Control sera were segregated into diagnosed negative regional endemics (*n* = 14, black) and non-endemic (*n* = 28, orange, sample origin Bangladesh); we note that there was greater discrimination between cases when using the non-endemic control sera. The sensitivity of the assay was 51.67% and specificity was 97.62%. Using the non-endemic controls alone, the sensitivity of the assay was 58% and the specificity was 96%. Using the endemic controls, the sensitivity was 50% and the specificity was 92%.

It is known that the popular serological test based on the rK39 antigen exhibits lower sensitivity in certain endemic regions including Brazil and East Africa (43), and so, we therefore sought to test the performance of our antigens to VL patient sera from different endemic regions. We first obtained diagnosed patient samples from Kenya and tested them against a selected panel of the same proteins from our library. Of these samples in this cohort, the majority were positive for rK39 (90.9%) and rK28 (88.6%). We again observed that the response to LdBPK_323600.1 (D36) gave the best diagnosis of infection status, although with reduced sensitivity compared with the Bangladesh cohort and the original diagnostic tests (Fig. 7).

**Fig 8:**
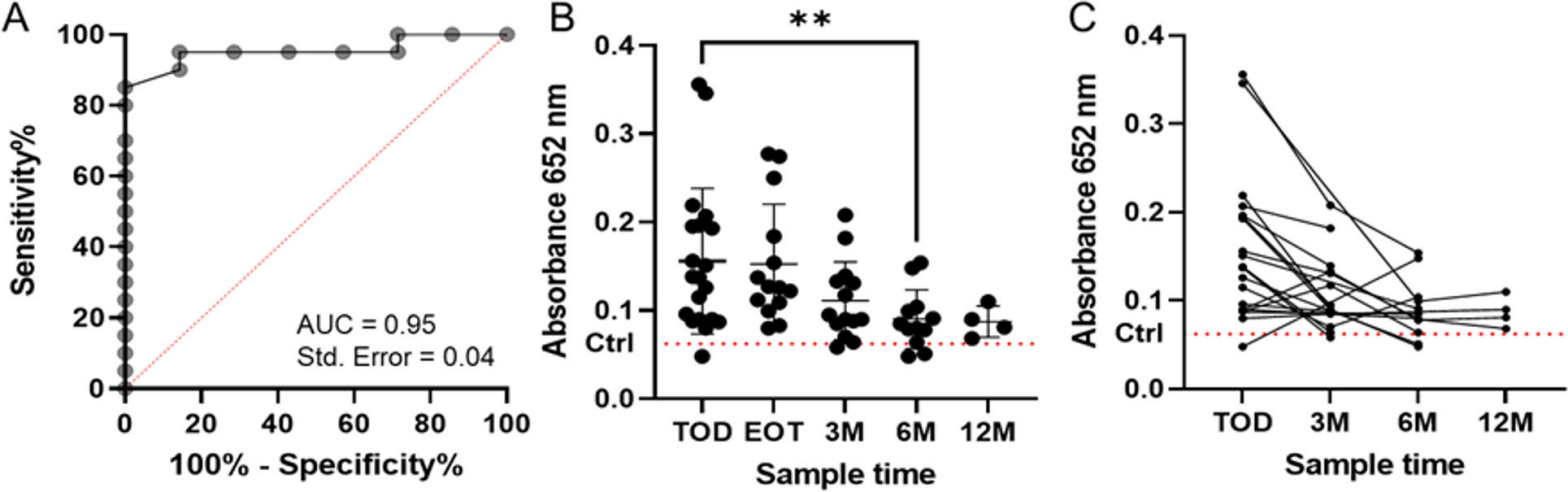
Immunoreactivity to the LdBPK_323600.1 (D36) antigen diagnosed infection which declines following treatment in Ethiopian patients. (**A**) Immunoreactivity to the LdBPK_323600.1 (D36) antigen from diagnosed Ethiopian visceral leishmaniasis patients (*n* = 20) relative to non-endemic controls (*n* = 7) represented as a receiver-operator curve (95% sensitivity, 86% specificity). (**B**) Immunoreactivity to recombinant LdBPK_323600.1 (D36) in individual patients was quantified at the time of diagnosis (TOD), end of treatment (EOT), and the 3, 6, and 12 months post-treatment. Data points are individual patients; bars represent mean ± SD. A one-way ANOVA found a significant difference between the means (*P* = 0.0153); subsequent pairwise comparisons were made using the Mann-Whitney test (**P < 0.01) finding a significant difference between the TOD and 6 months post-treatment. (**C**) Immunoreactivity to LdBPK_323600.1 (D36) declines within patients following treatment. Linked data points illustrate trends within individual patients. Dotted red line in panels **B** and **C** represents the mean immunoreactivity (*n* = 7) of non-endemic control samples.

Having established that serological responses to the LdBPK_323600.1 (D36) antigen were able to diagnose exposure to infection in patients from both the Indian subcontinent and East Africa, we next used a patient cohort from Ethiopia. Again, immunoreactivity to the D36 antigen classified infection with high sensitivity (95%) and specificity (86%) (Fig. 8A). Patients who were diagnosed as positive in the Ethiopian cohort were treated, and, where samples were available, patient-matched serum samples were taken again at 3, 6, and 9 months post-treatment. We quantified longitudinal antibody responses to LdBPK_323600.1 in the follow-up samples to determine if seroreactivity could be used as a marker of treatment success. We observed that in general, antibody titers to LdBPK_323600.1 declined after 3 months and showed a marked reduction in immunoreactivity after a year (Fig. 8B and C). Together, these data demonstrate that immunoreactivity to the LdBPK_323600.1 antigen can be used to diagnose visceral leishmaniasis infection in patients from different geographical locations and could be used to establish the success of treatment after 12 months.

## DISCUSSION

Visceral leishmaniasis poses a significant threat to the millions of individuals living in endemic regions and particularly during periods of civil unrest caused by social, economic, and natural disasters (44–46). There are increasing concerns that global warming will widen the geographical spread of the sand fly vectors (47–50), leading to millions more people living at risk of contracting this disease. As there are no licensed vaccines available for preventing infection and because the symptoms are similar to those for several other diseases, it is important to be able to accurately diagnose infections in endemic regions so that patients can receive correct and appropriate treatment.

It is important that any diagnostic test should be cost effective and deployable in the field within endemic regions. These requirements have favored the use of serological tests which include the DAT and detection of the rK39 antigen; however, both of these tests have limitations: the DAT requires access to a laboratory-like environment, and the rK39-based assay has varying sensitivity and specificity depending upon the endemic region (51–53). These limitations mean that better and more deployable diagnostics will be valuable tools for managing leishmaniasis. The genome of *L. infantum* was published over a decade ago (18) and offered the promise of identifying new diagnostic markers, and yet, few approaches have directly capitalized on these advances and investment. Our genome-centered approach described here identified several potential markers which, because of the possibility of patient serological responses varying between individuals, meant that sensitivity and specificity could potentially be improved by combining the responses to multiple antigens.

*Leishmania* parasites have a broad host range, and we were able to obtain sera from diagnosed infected mice, dogs, and humans. Although there were some differences, we generally observed that the same proteins were immunoreactive irrespective of the host species for both *L. donovani* and *L. infantum*. Indeed, D1 (LdBPK_290860.1), D36 (LdBPK_323600.1), D46 (LdBPK_362700.1), and D80 (LdBPK_220290.1) were immunoreactive across all three hosts and both species of parasite responsible for VL. We were not able to identify any simple biological feature common to these antigens such as relative protein abundance from publicly available data to provide a possible explanation for this. Whatever the biological reason for this concordance in immunoreactivity between these host species, this finding may aid in the development of a test that is suitable for both *L. donovani* and *L. infantum* in different hosts and endemic regions.

One other consideration for a serological marker is the extent to which the protein sequence varies between different parasite isolates. Immunoreactive proteins that are targets of the host antibody response are likely to be under diversifying selection which can lead to the problem of differences in diagnostic specificity in geographically distinct endemic regions as has been observed for the rK39 test. While the proteins within our panel are generally well conserved, even between the two recognized distinct species *L. donovani* and *L. infantum*, the gene encoding some of these proteins do show polymorphisms in parasite population sequencing studies (Table S5). Within the set of immunoreactive proteins described here, D46 (*LdBPK_362700.1*), D63 (*LdBPK_100680.1*), and D80 (*LdBPK_220290.1*) appear to be subjected to purifying selection (Ka/Ks ratios < 1), and D40 (*LdBPK_310530.1*) and D36 (*LdBPK_323600.1*) are under diversifying selection. These are small sample sizes, and so, it is currently unclear how this would impact their use as diagnostic antigens in different geographical regions. For example, there are only six known non-synonymous variants in *LdBPK_323600.1* (D36), which, in an antigen containing 533 amino acids, seems unlikely that these variants would significantly affect its immunoreactivity in diagnostic assays.

Although we report that several of the same antigens were immunoreactive in different hosts, there were intriguing differences, for example, immunoreactivity to D4 was only observed in *L. infantum* infection of dogs and humans and positive reactivity to D40, 63, and 82 was only identified in experimentally infected mice. These observations could be because mice were unnaturally infected by needle challenge or differences in either antigen presentation or pathology of infection between *L. donovani* and *L. infantum*.

One drawback of the current serological tests for VL is their inability to distinguish between current and historical infections due to the persistence of parasite-specific antibodies that can be present for many years after treatment (54) or even 2 years post-treatment with the rK39 test (55). Using pre- and post-treatment longitudinal sampling in Ethiopian patients, we could show that responses to D36 in most patients sero-reverted suggesting that response to this antigen could be used as a diagnostic of treatment success for longer term monitoring. There will likely be many challenges, however, in transferring this knowledge into a clinically useful diagnostic tool such as transferring the ELISA assay into a lateral flow format and tuning the tests to ensure that it retains a high level of accuracy. This may be particularly problematic if the levels of circulating anti-D36 antibodies are lower than the detection limit of the standard LFT secondary detection reagents. These levels would have to be empirically determined for each of the geographical regions where VL is present using fresh patient samples, something that we have been unable to test using only archival samples. This is important as it is not known how freeze-thaw cycles affect the recognition of the various antigens used in diagnostic tests, making an accurate comparison of the assay tests more challenging. It may be that our assay also suffers from regional variations, a problem that may be addressed by using multiple diagnostic antigens or a chimeric antigen containing multiple epitopes for diagnosis. The most accurate method for comparing these tests would be in a blinded trial at the point of diagnosis rather than using samples weighted toward a particular test metric that has varying sensitivity in different endemic regions. This study would also be improved by access to a larger longitudinal sampling cohort of patients from endemic regions, to determine if it may be a useful tool for surveillance and if the test may be useful in determining an infection before symptoms become prevalent.

From all the tested candidates, our attention was drawn to LdBPK_323600.1 (D36) because this was immunoreactive in all infected host species for both *L. donovani* and *L. infantum*, and responses gave high sensitivity and specificity in patient cohorts from several endemic regions. Structurally, D36 is a type I membrane protein containing an N-terminal signal peptide, transmembrane domain, and short cytoplasmic region. The extracellular region encodes a predicted alpha/beta hydrolase enzymatic activity although this provides few clues to its functional role as this fold is present in many enzymes with diverse functions including proteases and lipases (56). Orthologs of LdBPK_323600.1 are notably present in the genomes of trypanosomatid parasites capable of intracellular infections (*Leishmania spp*. and *T. cruzi*), suggesting that it has been lost from extracellular parasites such as *T. brucei*. Proteomics data suggest that the protein is present in both promastigote and amastigote because at least three unique peptides matching LdBPK_323600.1 were identified in studies of both stages of the life cycle (25). We do know from our own studies that the gene encoding LdBPK_323600.1 is not essential for infection—at least in a murine host—because parasites deficient in this gene did not show any overt difference in infection compared with parental controls (42); however, we cannot rule out its retention being advantageous for adaptation in the insect host stages of development between sandflies and reduviid bugs.

By capitalizing on the investments made in *Leishmania* genome sequencing, here, we have created a panel of recombinant antigens and systematically tested them as serological markers of infection. We have found that several antigens, and in particular LdBPK_323600.1, could be usefully included as part of improved diagnostic tools for this infectious tropical disease which threatens the lives of people living in many impoverished regions of the world.

## MATERIALS AND METHODS

### Ethics statements

#### Murine experiments

All murine experiments were performed under UK Home Office licence (PD3DA8D1F) and approved by the local Animal Welfare and Ethics Review Bodies. Mice used in the study were bred at the Research Support Facility at Wellcome Sanger Institute.

#### Pooled patient sera

To create the pools of human *L. donovani* and *L. infantum* infection, serum samples from the WHOCCL-ISCIII collection on leishmaniasis, registered at the National Biobank Register, Section Collections, Spain, with the collection Reference ID: C.0000898, were used (9). *L. infantum* samples were obtained from Spanish and the *L. donovani* samples from Ethiopian patients. Uninfected control human sera were collected from blood donors in the framework of a *Leishmania* infection prevalence study carried out in the Blood Bank of the Hospital of Fuenlabrada (Spain) approved by the Clinical Research Ethics Committee of the hospital with reference Apr 14-64.

#### Patient samples from Bangladesh

Sample collection was approved by the Ethical Review Committee of the International Centre for Diarrheal Disease Research, Bangladesh (icddr,b) (PR-14093), with adult participants providing written informed consent and parents/legal guardians of participants under 18 years of age providing informed consent on their behalf. Samples were collected from patients presenting with VL in addition to neighboring contacts in adjacent households as described elsewhere and classified using direct agglutination test, rK39 rapid diagnostic test (RDT), ELISA, pathological analysis, and PCR-based methods (57, 58).

#### Patient samples from Kenya

This study was approved by the Scientific and Ethics Review Unit (SERU) of the Kenya Medical Research Institute (KEMRI) Protocol (KEMRI/SERU/CCR/259/4483). Archived serum previously obtained from microscopy confirmed visceral leishmaniasis cases at Kacheliba Sub-County Hospital (West Pokot County) and Kimalel Sub-County Hospital (Baringo County) under KEMRI protocol SSC#3006, and VL-negative (clinically and serologically negative) individuals were used. Written informed consent was obtained from each adult study participant, while a parental/legal guardian written informed consent was obtained for each underage study participant. In addition to a parental/legal guardian informed consent, an assent was also obtained from 13- to 17-year-old study participants.

#### Patient samples from Ethiopia

This study was approved by the Institutional Review Board of the University of Gondar (IRB, reference O/V/P/RCS/05/1572/2017), the National Research Ethics Review Committee (NRERC, reference 310/130/2018), and Imperial College Research Ethics Committee (ICREC 17SM480). Informed written consent was obtained from each patient and control.

#### Canine samples

Samples used to create the pool were obtained from *L. infantum* naturally infected dogs in the framework of a vaccine study approved by the Department of Biodiversity and the Environment of the Government of Catalonia under number 6760 in accordance with Spanish law on the protection of animals used for experimentation and other scientific purposes (Royal Decree 1201/2005 and Law 32/2007). The Spanish legislation is a transposition of Directive 86/609/EEC.

#### Individual samples

All dogs were enrolled in this retrospective study with informed consent from their caretakers, and all protocols followed were approved by the University of Iowa Institution Animal Care and Use Committee (IACUC) an AAALAC-accredited institution following the requirements for the US National Institutes of Health Office of Laboratory Animal Welfare Assurances which operates under the 2015 reprint of the Public Health Service Policy on Humane Care and Use of Laboratory Animals. Blood was collected in heparinized or tubes containing EDTA. Diagnosis of infection status was made using a quantitative polymerase chain reaction (qPCR)-based assay or a serological assay Dual Path Platform canine visceral leishmaniasis (DPP CVL Chembio Diagnostic Systems Inc., Medford, NY) designed to detect immunoreactivity to a recombinant kinesin rK28 or immunofluorescence-based assay details which have already been published elsewhere (59). Some canines received therapeutic treatment with the LeishTec vaccine, and all dogs were scored for clinical symptoms of canine leishmaniasis (enlarged lymph nodes, enlargement of the spleen) (59).

### Library of *L. donovani* cell surface and secreted proteins

Sequences encoding extracellular domains excluding the transmembrane-spanning region and signal peptide (Table S1) were codon optimized for mammalian expression, synthesized (GeneartAG or Twist Bioscience), and cloned into a derivative of the pTT3 mammalian expression vector (60) lacking the Cd4 tag (61). Expression constructs were transiently co-transfected in HEK293E or HEK293-6E with a plasmid encoding a secreted BirA (62) which enzymatically monobiotinylated proteins with a C-terminal tag (63). Protein expression in culture supernatants was determined by western blotting using HRP-conjugated streptavidin (Pierce Bioscience). Expression constructs which did not yield high levels of protein were subcloned into an expression plasmid containing a C-terminal rat Cd4d3+4 tag in an attempt to improve expression (32). Proteins were purified from spent tissue culture supernatants using nickel affinity chromatography as described (61). Filtered culture supernatants were incubated with Nickel-NTA agarose (Jena Bioscience) and washed twice with binding buffer (20 mM sodium phosphate, 400 mM NaCl, 20 mM imidazole, and pH 7.4). Bound proteins were eluted using an elution buffer (20 mM sodium phosphate, 400 mM NaCl, 400 mM imidazole, and pH 7.4). Details of the protein library are provided in Table S1.

### *L. donovani* and *L. infantum* promastigote culture and animal infections

The luciferase-expressing strains of *L. donovani* and *L. infantum* (MHOM/BR/00/1669) (64) were cultured at 28°C in LdPro medium (65) supplemented with 100 µg/mL nourseothricin and 10 U/mL penicillin-streptomycin (Gibco). Wild-type (LV9; WHO designation: MHOM/ET/67/HU3) *L. donovani* were cultured in the same medium without nourseothricin supplementation. Parasite maintenance for virulence and infection parameters used *in vivo* were as previously described (35). Briefly, cultures were seeded at an initial density of 1 × 10^6^ cells/mL. After 7 days, stationary-phase promastigotes were harvested by centrifugation (2,000 × *g*, 10 min), washed twice with PBS, and resuspended at 5 × 10^8^ parasites per mL in DMEM medium (Sigma). Female BALB/c mice were infected intravenously using 200 µL/injection/animal inoculum. Infection progression in livers and spleens was monitored by bioluminescent *in vivo* imaging at regular intervals as previously described (35).

### Enzyme-linked immunosorbent assay

Purified recombinant proteins were normalized before use in ELISA studies as previously described (21, 22). Briefly, purified biotinylated proteins were diluted in blocking buffer (HEPES-buffered saline containing 0.05% [vol/vol] Tween 20 [HBST] and 2% [wt/vol] BSA) were immobilized in the wells of streptavidin-coated microtiter plates. After three washes using HBST, sera were diluted 1:1,000 in blocking buffer and applied to the wells and incubated for 1 h at room temperature. Immunoreactivity was quantified using the appropriate species-specific-conjugated alkaline phosphatase (AP)-conjugated, horseradish peroxidase (HRP)-conjugated, or species-specific secondary antibodies (goat anti-canine IgG LSBio C60297, goat anti-mouse IgG, anti-human IgA IgG, and IgM Jackson 109-035-064) and Sigma-104 alkaline phosphatase substrate (1 mg/mL, 100 µL) or 3,3′,5,5′-tetramethylbenzidine (TMB) (Invitrogen).

### Statistical analysis

Statistical analysis was performed in GraphPad Prism version 9.5.1. Receiver operator curves were generated from the ELISA data, with the confidence intervals of the AUC reported at 99%.

## Data Availability

Expression plasmids encoding the proteins described here are available from Addgene using the identifiers provided in Table S1.

## SUPPLEMENTAL MATERIAL

Figure S1 mbio.00859-24-s0001.tif The immunoreactivity to the library of recombinant soluble *Leishmania* proteins is heat labile.

Legends mbio.00859-24-s0002.docx Supplemental material legends.

Supplemental Tables mbio.00859-24-s0003.xlsx Tables S1 to S5.

ASM does not own the copyrights to Supplemental Material that may be linked to, or accessed through, an article. The authors have granted ASM a non-exclusive, world-wide license to publish the Supplemental Material files. Please contact the corresponding author directly for reuse.
