# Supplementary figures and images for: A panel of recombinant Leishmania donovani cell surface and secreted proteins identifies LdBPK_323600.1 as a serological marker of symptomatic infection
Source: mBio. 2024 Apr 19;15(5):e00859-24. doi: 10.1128/mbio.00859-24 (PMC11077996; doi:10.1128/mbio.00859-24)

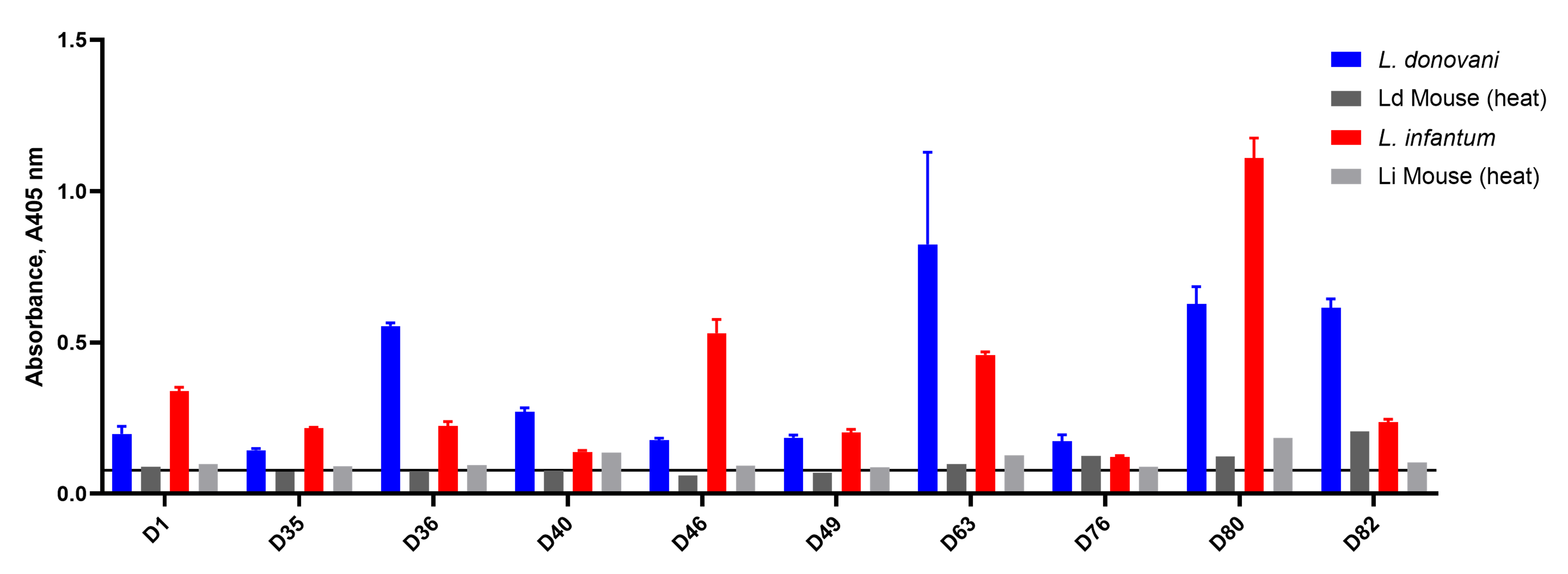

Supplement: Figure S1 — The immunoreactivity to the library of recombinant soluble Leishmania proteins is heat labile. [file mbio.00859-24-s0001.tif]
