# Supplementary material for: A panel of recombinant Leishmania donovani cell surface and secreted proteins identifies LdBPK_323600.1 as a serological marker of symptomatic infection
Source: mBio. 2024 Apr 19;15(5):e00859-24. doi: 10.1128/mbio.00859-24 (PMC11077996; doi:10.1128/mbio.00859-24)
Supplement: Legends — Supplemental material legends. [file mbio.00859-24-s0002.docx]

**Supplemental Figure Legend**

**Figure S1. The immunoreactivity to the library of recombinant soluble *Leishmania* proteins is heat labile.** Enzymatically biotinylated *Leishmania* recombinant proteins that displayed immunoreactivity greater than 2 SD above the mean of naive sera were immobilised on streptavidin-coated microtitre plates after heat treatment (80°C for 10 mins) or left untreated. The immunoreactivity to pooled immune serum from chronically-infected mice was quantified by ELISA. Bars represent means ± SD where blue/dark grey = *L. donovani* serum untreated/heat treated; red/light grey = *L. infantum* serum untreated/heat treated. The black line represents the mean measurement of naive sera against the whole protein library to uninfected control mouse serum.

**Supplemental Table legends**

**Table S1. The library of *Leishmania donovani* proteins.** A table listing the properties of each member of the *L. donovani* protein library. Each protein is given a systematic “D” number with their accession numbers (*L. donovani* and *L. infantum* orthologue), protein name and Addgene plasmid number. Amino acid sequences of *L. donovani* proteins were analysed using prediction software tools (TMHMM 2.0 and SignalP 4.0), except for D82 where the amino acid sequence of the *L. infantum* protein was used. Protein architectures were classified as either “Secreted”, “Type I”, “GPI anchor”, or “Multipass”. Successful expression of the protein is indicated and in which HEK cell type and whether this required the rat Cd4d3+4 tag. The number of theoretical N-linked glycosylation sequons is indicated together with the predicted mass corrected for the presence of the Cd4 tag and mass of the average N-linked glycan. The ectodomain protein sequence is also provided.

**Table S2. Clinical metadata for the dogs in the disease progression group.** Clinical metadata is provided for each dog within the clinical progression group along with basic information on sex, age and whether the dog was vaccinated or not (placebo). A numerical value where a higher number indicates more advanced disease progression is provided, along with a physical examination (PE) description of the pathological symptoms of the animals in the disease progression group at 9 months.

**Table S3. Serological immunoreactivity of canine sera to a subpanel of antigens.** Soluble recombinant enzymatically monobiotinylated *L. donovani* antigens identified by their “D number” were immobilised in individual wells of a streptavidin-coated plate and the immunoreactivity to sera from vertically-infected dogs after having received a therapeutic vaccine or placebo was quantified by ELISA. Individual identified animals were grouped according to whether disease progressed (P) or regressed (R), and whether they had been vaccinated (Y), or not (N). A numerical clinical score is provided from a physical examination at 9 months where a higher number indicates more advanced disease. Numerical values are background subtracted ELISA absorbance values.

**Table S4. Clinical metadata associated with serum samples analysed from Bangladesh and Kenya.** Clinical metadata including geographical origin, sample group, age, gender are provided. In some cases, information was not available (NA).

**Table S5. Genetic polymorphisms in target antigens for serological markers of visceral leishmaniasis.** The number of synonymous (Syn) and non-synonymous (Non-Syn) polymorphisms identified in the genes encoding immunoreactive antigens were obtained from the TriTrypDB, and their ratio (Ka/Ks) calculated.
